# High Discharge Energy Density at Low Electric Field Using an Aligned Titanium Dioxide/Lead Zirconate Titanate Nanowire Array

**DOI:** 10.1002/advs.201700512

**Published:** 2017-12-27

**Authors:** Dou Zhang, Weiwei Liu, Ru Guo, Kechao Zhou, Hang Luo

**Affiliations:** ^1^ State Key Laboratory of Powder Metallurgy Central South University Changsha 410083 Hunan Province China

**Keywords:** ceramics, composites, dielectric materials, discharge energy density, nanowire arrays

## Abstract

Polymer‐based capacitors with high energy density have attracted significant attention in recent years due to their wide range of potential applications in electronic devices. However, the obtained high energy density is predominantly dependent on high applied electric field, e.g., 400–600 kV mm^−1^, which may bring more challenges relating to the failure probability. Here, a simple two‐step method for synthesizing titanium dioxide/lead zirconate titanate nanowire arrays is exploited and a demonstration of their ability to achieve high discharge energy density capacitors for low operating voltage applications is provided. A high discharge energy density of 6.9 J cm^−3^ is achieved at low electric fields, i.e., 143 kV mm^−1^, which is attributed to the high relative permittivity of 218.9 at 1 kHz and high polarization of 23.35 µC cm^−2^ at this electric field. The discharge energy density obtained in this work is the highest known for a ceramic/polymer nanocomposite at such a low electric field. The novel nanowire arrays used in this work are applicable to a wide range of fields, such as energy harvesting, energy storage, and photocatalysis.

Energy storage materials play an indispensable role in modern electronic devices and electric power systems. The development of high‐energy‐storage‐density devices is of critical importance to meet the ever‐increasing demand for these applications.^1^, ^2^ Dielectric materials, which possess high charge–discharge capability to store and release electrical energy through dielectric polarization and depolarization, have attracted immense interest for potential applications in energy storage devices, such as capacitors.^3^ The discharge energy density (*U*_dis_) of a dielectric material is given by^4^

(1) $$U_{\mathrm{dis}}=\int EdD$$

where *E* is the applied electric field and *D* is the electric displacement

(2) $$D=\varepsilon_{0}\varepsilon_{r}E$$

where ε_0_ and ε_r_ are vacuum permittivity and the relative permittivity of the dielectric, respectively. Therefore, relative permittivity and breakdown strength are key factors in determining the energy density.

Among numerous dielectric materials, polymer nanocomposites are receiving growing attention because of their advantage in combining the merits of ceramics (e.g., high permittivity) with those of polymers (e.g., high breakdown strength).^2^, ^5^ A number of studies have reported ceramic/polymer nanocomposites with high energy density.^6^, ^7^ For example a discharge energy density of 20 J cm^−3^ at 646 kV mm^−1^ was reported for BaTiO_3_@TiO_2_ core–shell fibers in a polyvinylidene fluoride polymer matrix (denoted as BaTiO_3_@TiO_2_/PVDF, where @ denotes a core–shell structure).^8^ This was subsequently improved to 31.2 J cm^−3^ at 797.7 kV mm^−1^ for nanocomposites with large aspect ratio fibers as a result of their preferred orientation directions perpendicular to the external electric field.^9^ However, these nanocomposites still possess relatively low energy density at a lower electric field, e.g., an energy density of ≈2.8 J cm^−3^ at the electric field of 250 kV mm^−1^. Use of these materials exclusively at high applied electric field is likely to present challenges relating to the failure probability. More recently, it was shown by Xie et al. that a high discharge energy density of 10.8 J cm^−3^ at a relatively low electric field of 240 kV mm^−1^ could be achieved in BaTiO_3_/PVDF nanocomposites with aligned BaTiO_3_ nanowires.^10^

Therefore, polymers incorporated with aligned ceramic nanowires represent an effective design for high discharge energy density systems.^10^, ^11^ However, the preparation of aligned ceramic nanowires is challenging, especially for aligned ferroelectric ceramic nanowire arrays, such as lead zirconate titanate (PZT) and BaTiO_3_ nanowire arrays due to their randomly oriented grains.^12^ A simple method to prepare high‐performance capacitors utilizing anatase TiO_2_ nanowire arrays as the fillers has recently been reported.^13^, ^14^ However, TiO_2_ is not a ferroelectric and has relatively low permittivity and low saturation polarization, which lead to limited energy density.

PZT is the most widely used piezoelectric ceramic and shows advantages in energy storage applications attributed to the high permittivity, low remanent polarization, and low cost. Combining the electrical properties of PZT with TiO_2_ to readily form nanowires represnts an attractive strategy for the fabrication of a high discharge energy density nanocomposite. Here we present a simple two‐step method for synthesizing titanium dioxide/lead zirconate titanate (TiO_2_@PZT) nanowire arrays; the PZT was designed as a shell layer coated on the surface of TiO_2_ nanowires via sol spin‐coating and subsequent annealing. The morphologies of the synthesized TiO_2_@PZT nanowire arrays were tuned by modulating the concentration of the Ti source. Poly(vinylidene fluoride‐trifluoroethylene‐chlorotrifluoroethylene) (P(VDF‐TrFE‐CTFE)) was selected as the polymer matrix to combine with the synthesized TiO_2_@PZT nanowire arrays, since it is a relaxor ferroelectric polymer with high permittivity and low remnant polarization.^15^ Due to the high interfacial polarization, nanocomposites with high permittivity and high discharge energy density at low electric field were achieved. This work provides a novel route to prepare ceramic nanowire arrays and high energy density polymer based capacitors capable of operating at low voltage.

**Figure** **1** shows the X‐ray diffraction (XRD) patterns of the TiO_2_ nanowire array, Pb(Zr_0.52_Ti_0.48_)O_3_ powders, and the TiO_2_@PZT nanowire arrays. As illustrated in Figure 1a, in addition to the peaks of the fluorine doped tin oxide (FTO) substrate, three peaks located at 2θ values of 36.30°, 54.69°, and 63.11° are observed, which match well with the (101), (211), and (002) planes of rutile TiO_2_ (JCPDS No. 01‐88‐1172).^13^ The narrow peak width indicates that TiO_2_ nanowires possess relatively good crystallinity. The XRD pattern of the PZT power derived from the sol confirms PZT to be in the perovskite tetragonal phase with lattice constants of *a* = 0.4055 nm and *c* = 0.4110 nm, matching well with the literature values of *a* = 0.4036 nm and *c* = 0.4146 nm (JCPDS No. 33‐0784). The XRD pattern of TiO_2_@PZT nanowire array can be observed in Figure 1b. In addition to the strong peaks associated with TiO_2_, there are weaker peaks at 30.92°, 31.36°, and 44.88° 2θ degrees, corresponding to the (101), (110), (200) planes of PZT.

**Figure 1:**
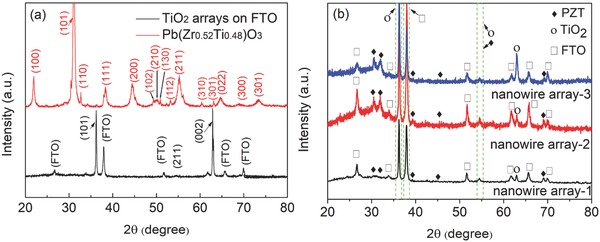
a) XRD patterns of TiO_2_ nanowire array and Pb(Zr_0.52_Ti_0.48_)O_3_ powders; b) XRD patterns of the TiO_2_@PZT nanowire arrays.

**Figure** **2**a,b illustrates the cross‐sectional and the surface scanning electron microscope (SEM) images of the TiO_2_ nanowire array grown on FTO, respectively. It can be observed that the TiO_2_ nanowire array is uniformly distributed on the FTO substrate and had preferred orientation in the [001] direction. It is believed that FTO substrate plays an important role in generating the 1D structure by promotion of epitaxial nucleation and growth in the [001] direction due to the small lattice mismatch between the rutile structures of FTO and TiO_2_.^16^ The morphologies of the synthesized TiO_2_@PZT nanowire arrays were tuned by modulating the concentration of Ti source from 0.03 to 0.06 mol L^−1^, as shown in Figure S1 in the Supporting Information. After the spin‐coating and annealing process, the PZT phase was coated on the surface of TiO_2_ nanowire array. The resulting TiO_2_@PZT nanowire arrays were relatively denser than the parent TiO_2_ nanowire array, as shown in Figure 2d, and maintained the perpendicular orientation to the substrate.

**Figure 2:**
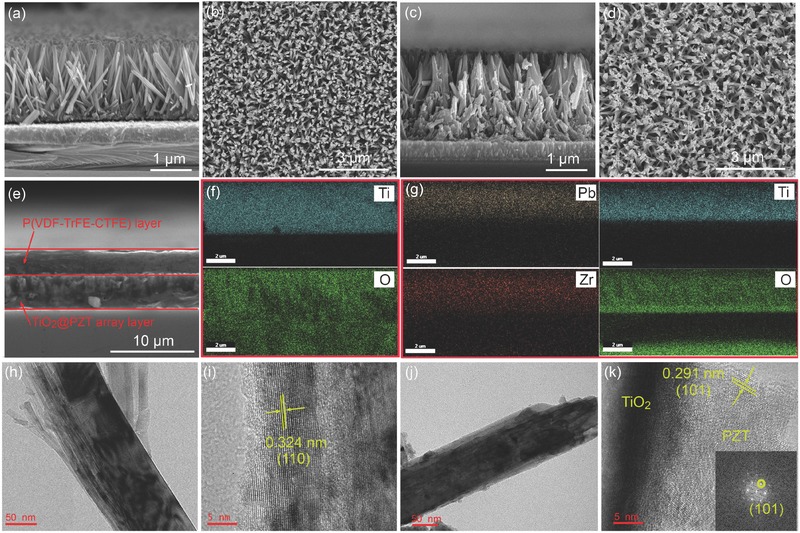
SEM images of TiO_2_ nanowire array grown on FTO: a) the cross‐sectional view and b) the top view. SEM images of TiO_2_@PZT nanowire array: c) the cross‐sectional view and d) the top view. e) The cross‐sectional view of the TiO_2_@PZT nanowire array/P(VDF‐TrFE‐CTFE) nanocomposite. f) SEM image of TiO_2_ nanowire array, the corresponding energy‐dispersive X‐ray spectroscopy (EDS) elemental mapping for Ti and O. g) SEM image of TiO_2_/PZT nanowire array, the corresponding energy‐dispersive X‐ray spectroscopy (EDS) elemental mapping for Pb, Zr, Ti, and O. h,i) TEM image and HRTEM images of TiO_2_ nanowires. j,k) TEM image and HRTEM images of TiO_2_@PZT nanowires.

Figure 2e shows an image of the TiO_2_@PZT nanowire array‐2 polymer nanocomposite, which was typical and was ≈7 µm thick. The upper P(VDF‐TrFE‐CTFE) layer had a thickness of ≈4 µm, and the TiO_2_@PZT nanowire array can be seen sandwiched between the polymer layer and the FTO substrate. No obvious defects were found in the nanocomposites, which indicated that the TiO_2_/PZT nanowire array was tightly encapsulated by the polymer matrix. Energy‐dispersive X‐ray spectroscopy (EDS) elemental mapping and transmission electron microscopy (TEM) results in Figure 2c–k were used to prove the PZT layer was coated on the surface of TiO_2_ nanowires. Ti and O are seen to be uniformly distributed in the TiO_2_ nanowire array (Figure 2f), while in the TiO_2_@PZT nanowire array shown in Figure 2g Pb and Zr were additionally detected in the EDX map. Figure 2h shows a TEM image of the as‐prepared TiO_2_ nanowires illustrating a clear nanowire shape with a diameter of 10–80 nm. The high‐resolution transmission electron microscopy (HRTEM) image of the TiO_2_ nanowire is depicted in Figure 2i, with a lattice spacing of 0.324 nm, which matches well with the (110) diffraction plane of rutile TiO_2_. Figure 2j shows the TEM image of a TiO_2_@PZT nanowire, indicating the existence of a thin layer on the fringe of TiO_2_ nanowire. Figure 2k shows an HRTEM image of this thin layer, which shows a lattice spacing of 0.291 nm attributable to the (101) plane of the tetragonal phase of PZT, consistent with the XRD results. These results confirm the successful preparation of TiO_2_@PZT nanowire arrays.

**Figure** **3**a,b shows the variation of the permittivity and dielectric loss of P(VDF‐TrFE‐CTFE) and TiO_2_@PZT nanowire array/P(VDF‐TrFE‐CTFE) nanocomposites with different concentrations of the Ti source as a function of frequency at room temperature. As illustrated in Figure 3a, the permittivity of the nanocomposites decreases with increasing frequency for all samples, and increases with increasing Ti source concentration over the whole frequency range. The permittivity of the TiO_2_@PZT nanowire array‐1 nanocomposite reached 168.7 at 1 kHz, while that of the pure P(VDF‐TrFE‐CTFE) sample was 33.5 at the same frequency. The permittivities of the other two samples were 198.3 and 218.9 at 1 kHz, respectively. All nanocomposites showed much higher relative permittivity than the pure polymer. This can be explained by considering the various contributions to the overall polarization made by the different components of the composite and the interfaces between them. First, the aligned TiO_2_ nanowire array fillers play an important role in contributing to the polarization. Yao et al. reported that nanocomposites with orientated TiO_2_ nanowire array fillers showed significantly enhanced permittivity.^13^ In addition the PZT has a high relative permittivity, with values around 1800,^17^ while the terpolymer P(VDF‐TrFE‐CTFE) itself also has a relatively high permittivity, which is demonstrated by the test in Figure S2 (Supporting Information). Second, the system comprises two large interfacial regions, i.e., the TiO_2_‐PZT and PZT‐P(VDF‐TrFE‐CTFE) interfaces. The interfacial polarizations will also contribute to the enhanced permittivity of the system.

**Figure 3:**
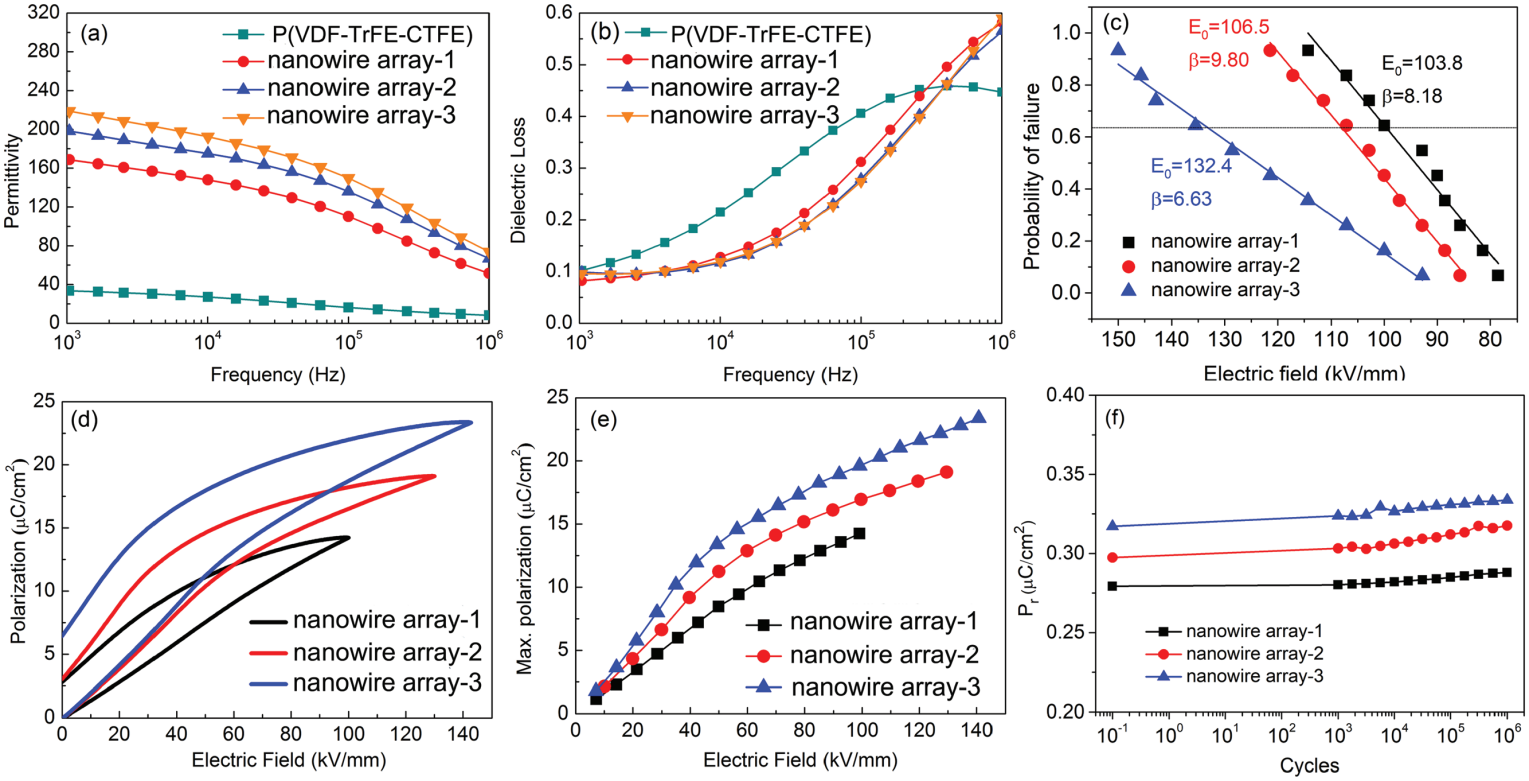
Dependences of a) permittivity and b) dielectric loss on frequency for neat polymer and nanocomposites; c) breakdown strength, d) typical *P–E* loops, and e) the maximum dielectric displacement measured from the nanocomposites; f) endurance test of the nanocomposites with an electric field cycling amplitude of 40 kV mm^−1^.

The breakdown strengths of the nanocomposites analyzed with a two‐parameter Weibull distribution function are shown in Figure 3c. As can be seen, the characteristic breakdown strengths are relatively lower than many reported results. There are a number of reasons why breakdown strengths are lower than in other nanocomposite systems; these include (i) defects at the interfaces between components of the composites can lead to premature failure; (ii) the electric field is mainly contained in the thin polymer layer above the nanowire array due to the big permittivity contrast between the nanowire array and polymer matrix; and (iii) the relatively high conductivity and leakage current shown in Figures S3 and S4 in the Supporting Information can also decrease the breakdown strength of the composites due to the increased charge motion.^18^ Typical electrical polarization–electric field (*P–E*) loops of the nanocomposites measured at a frequency of 10 Hz at room temperature are shown in Figure 3d. The maximum polarization (*P*_max_) values for the TiO_2_@PZT nanowire array‐1, TiO_2_@PZT nanowire array‐2, and TiO_2_@PZT nanowire array‐3 nanocomposites were 14.22, 18.38, and 23.35 µC cm^−2^, respectively (Figure 3e), while that for the pure terpolymer was 5.2 µC cm^−2^.^19^ The increase of *P*_max_ with increasing Ti concentration is in agreement with our previous work,^14^ which also indicated that the density and orientation of TiO_2_ nanowire array play an important role in the enhancement of dielectric displacement for the nanocomposites. The PZT phase introduced to the nanocomposites provides more interfacial areas; therefore, the enhanced interfacial polarization leads to a high total polarization value. The relation between electric displacement (*D*) and polarization (*P*) is given by

(3) $$D=\varepsilon_{0}E+P$$

The maximum in electric displacement, *D*_max_, is approximately equal to *P*_max_ due to the low value of the vacuum permittivity. The difference between *P*_max_ and residual polarization (*P*_r_) for the TiO_2_@PZT nanowire array‐1 nanocomposite reached 11.42 µC cm^−2^ at 100 kV mm^−1^, while that for the TiO_2_@PZT nanowire array‐2 nanocomposite was 15.62 µC cm^−2^ at 130 kV mm^−1^, the highest value of 16.93 µC cm^−2^ achieved for the TiO_2_@PZT nanowire array‐3 nanocomposite at an electric field of 143 kV mm^−1^. Indeed, the *P*_max_ and *P*_max_ − *P*_r_ values for the TiO_2_@PZT nanowire array‐3 nanocomposite are the highest recorded at such a low electric field for a polymer‐based dielectric. It is worth noting that the enhanced *P*_max_ and *P*_max_ − *P*_r_ values at a low electric field are critical for obtaining a high‐endurance high‐discharge energy density. High fatigue endurance is an important factor during the long‐term charge–discharge cycling process of dielectrics. Charge–discharge cycling was carried out for up to 10^6^ cycles, with pulse field amplitudes of 40 kV mm^−1^, four points per decade of 4, and a pulse frequency of 1 kHz. The residual polarization (*P*_r_) of the nanocomposites as a function of the cycle number is shown in Figure 3f. It can be seen that the nanocomposites hold high fatigue endurance, e.g., the *P*_r_ of the TiO_2_@PZT nanowire array‐3 nanocomposite only increased by 6.8% after 10^6^ cycles.

**Figure** **4**a shows a typical *P–E* loop for a nonlinear dielectric material, in which the blue colored region represents the discharge energy density, the red region is the energy loss, and the stored energy density includes the discharge energy density and energy loss. The discharged energy density and efficiencies for the TiO_2_@PZT nanowire array nanocomposites were determined from their *P–E* loops and are plotted as a function of electric field in Figure 4b–d. The efficiency, η, was calculated as follows

(4) $$\eta =U_{\mathrm{dis}}/U_{\mathrm{stor}}$$

where *U*_stor_ is the energy storage density. All of the nanocomposites exhibit a large discharge energy density and a relatively high η value. The maximum discharge energy density of TiO_2_@PZT nanowire array nanocomposites was 4.0 J cm^−3^ (η = 58.1%) at 100 kV mm^−1^ and 6.0 J cm^−3^ (η = 59.7%) at 130 kV mm^−1^, arrays 1 and 2, respectively. Most notably, the TiO_2_@PZT nanowire array‐3 nanocomposite achieved the highest discharged energy density of 6.9 J cm^−3^ with a relatively high η of 50.0% at an electric field at 143 kV mm^−1^. To the best of our knowledge, this is the highest discharge energy density obtained at such a low electric field for a polymer‐based nanocomposite. **Table** **1** summarizes the performance of some typical polymer‐based dielectric composites with the electric field. The large energy density at such a low electric field in these nanocomposites can be attributed to (i) the dual phase nature of the TiO_2_@PZT nanowire array, which results in large interfacial regions and hence large interfacial polarization;^24^, ^25^ (ii) the highly oriented nanowire also plays an important role in increasing the polarization and electromechanical coupling of the nanocomposites;^6^, ^16^ and (iii) the obtained high permittivity of the nanocomposites yields high saturation polarization (Equation (3)), and the nanocomposites possess narrow hysteresis loops with low values of *P*_r_ as shown in Figures S5–S7 (Supporting Information), which lead to largely enhanced *P*_max_ − *P*_r_ values.

**Figure 4:**
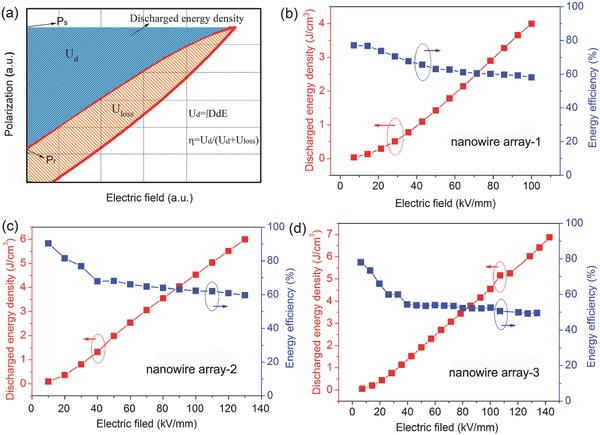
a) Electric polarization‐electric field (*P–E*) loop. b–d) Energy density and efficiency as a function of the electric field for TiO_2_@PZT nanowire array/P(VDF‐TrFE‐CTFE).

**Table 1:** Comparison of discharged energy density for dielectric composites with different nanofiber fillers at applied electric field

Matrix	Fillers	*E*_b_ [kV mm^−1^]	*U*_dis_ [J cm^−3^]	*E* [kV mm^−1^]	*U*_dis_ [J cm^−3^]	Ref.
PVDF	BaTiO_3_	470	18.8	200	≈3.8	6
PVDF	BaTiO_3_@TiO_2_	350	12. 5	150	≈2.8	20
P(VDF‐HFP)	BaTiO_3_@TiO_2_	797.7	31.2	200	≈2.4	9
PVDF	BaTiO_3_	450	10.0	174	≈2.6	21
PVDF	BaTiO_3_@Al_2_O_3_	420	10.6	200	≈2.2	22
PVDF	Ba_0.2_Sr_0.8_TiO_3_	450	14.9	150	≈2.0	7
P(VDF‐TrFE‐CFE)	BaTiO_3_	300	10.6	150	≈4.0	23
P(VDF‐TrFE‐CTFE)	TiO_2_@PZT	–	–	143	6.9	This work

It has been demonstrated that TiO_2_@PZT nanowire arrays can be prepared by a simple hydrothermal method via a spin‐coating and annealing process. The TiO_2_@PZT nanowire array polymer nanocomposites exhibit significantly enhanced permittivity, as well as electric displacement at low electric field, compared to pure polymers and most previously reported polymer nanocomposites. This is achieved due to high interfacial polarization and interfacial coupling effects. It has been shown that a TiO_2_@PZT nanowire array nanocomposite can achieve a high energy density of 6.9 J cm^−3^ at a low electric field of 143 kV mm^−1^. This work provides a promising strategy for high‐energy density capacitors at a lower operating voltage.

## Experimental Section

*Materials*: The terpolymer P(VDF‐TrFE‐CTFE) composition had a mole ratio of 63:29:8 and was provided by PolyK Technologies, LLC; FTO substrate (Pilkington, TEC7 coated, 1.6 mm thickness, 7 Ωsq^−1^), tetrabutyl titanate (C_16_H_36_O_4_Ti) (Guoyao from China, 98.0%), lead acetate trihydrate [Pb(CH_3_COO)_2_.3H_2_O] (Guoyao from China, 99.5%), zirconium nitrate pentahydrate [Zr(NO_3_)_4_.5H_2_O] (Guoyao from China), and tetrabutyl titanate (C_16_H_36_O_4_Ti). All chemicals were used “as received,” unless indicated.

*Synthesis of TiO_2_ Nanowire Array*: The TiO_2_ nanowire arrays were synthesized through a hydrothermal method. A solution containing a mixture of 25 mL HCl (36–38%), 25 mL deionized (DI) water, and various volumes of C_16_H_36_O_4_Ti (0.5, 0.7, and 1.0 mL corresponding to 0.03, 0.04, and 0.06 mol L^−1^, respectively) was stirred until it became transparent. Subsequently, the transparent solution was transferred into a 100 mL autoclave together with a piece of FTO substrate (10 mm × 15 mm), which was sonicated successively in acetone, ethanol, and DI water for 10 min. The autoclave was maintained at 180 °C for 3 h to synthesize the TiO_2_ nanowire array on FTO substrate.

*Preparation of the PZT Sol*: A mixed solution with a Pb:Zr:Ti mole ratio of 1.1:0.52:0.48 was prepared from appropriate portions of lead acetate trihydrate [Pb(CH_3_COO)_2_.3H_2_O], zirconium nitrate pentahydrate [Zr(NO_3_)_4_.5H_2_O], and tetrabutyl titanate [C_16_H_36_O_4_Ti]. At first, the lead acetate and zirconium nitrate pentahydrate was dissolved in 2‐methoxyethanol separately and then mixed together and stirred for 20 min. Due to the instability of tetrabutyl titanate, three to four drops of acetyl acetone were added to the solution as a stabilizer. Next, the solution was heated at 97 °C and stirred for 20 min. Finally, the tetrabutyl titanate was dropped into the solution, which was heated at 80 °C and stirred for 1 h. The lead zirconium titanium solution was aged for 1 week, resulting in a light yellow clear solution.

**Scheme 1:**
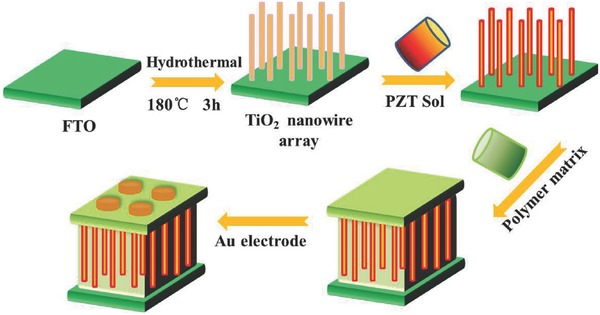
Preparation process of TiO_2_@PZT nanowire array/P(VDF‐TrFE‐CTFE) nanocomposite with gold electrodes.

*Preparation of Nanocomposites*: The TiO_2_ nanowire arrays on the FTO substrate were dipped into the Pb(Zr_0.52_Ti_0.48_)O_3_ (PZT, 0.2 mol L^−1^) sol, and treated by spin‐coating, followed by heating at 200 °C for 5 min, 350 °C for 5 min, 400 °C for 5 min, and then thermal annealing at 600 °C for 500 s in air. The P(VDF‐TrFE‐CTFE)/*N,N*‐dimethylformamide solution was coated on the surface of the TiO_2_@PZT nanowire arrays by spin‐coating to obtain the TiO_2_@PZT/P(VDF‐TrFE‐CTFE) nanocomposite with a thickness of ≈7 µm. The obtained samples were dried at 70 °C for 24 h. For electrical measurements the nanocomposites were sputtered with gold electrodes. **Scheme** **1** summarizes the preparation procedure for the TiO_2_@PZT/P(VDF‐TrFE‐CTFE) nanocomposites. The resulting nanocomposites were defined as TiO_2_@PZT nanowire array‐1, TiO_2_@PZT nanowire array‐2, and TiO_2_@PZT nanowire array‐3, for the nanowire arrays prepared from 0.03, 0.04, and 0.06 mol L^−1^ Ti precursor solutions, respectively

*Characterization*: The crystalline phases were characterized by XRD (Rigaku D‐Max/2550VB^+^) utilizing Cu K_α_ radiation (λ = 1.5418 Å). The morphology and alignment of the nanowire arrays were observed by SEM (JSM‐6390). TEM images of the samples were taken with a Titan G2 60‐300, using an accelerating voltage of 300 kV. Gold electrodes with a thickness of ≈20 nm were sputtered onto the nanocomposites (1.0 cm × 1.5 cm) using an eyelets mask with 2 mm diameter for electrical tests. The frequency dependence of permittivity and dielectric loss were performed using an Agilent 4294A LCR meter with a frequency range from 40 Hz to10 MHz at room temperature. The permittivity was calculated from the measured capacitance using the relation ε_r_ = *Cd*/ε_0_ *A*, where ε_r_ is the permittivity of the capacitor, *C* is the capacitance (Farads), *d* is the thickness (m) of the samples, ε_0_ is the permittivity of free space (8.854 × 10^−12^ F m^−1^), and *A* is the surface area of the capacitor's electrode (m^2^). The *P–E* loops of the nanocomposite were measured at 10 Hz at room temperature using a TF analyzer 2000 ferroelectric polarization tester (aixACT, Germany) and a Delta 9023 furnace in a silicone oil bath to avoid electrical discharges that would occur in air.

## Conflict of Interest

The authors declare no conflict of interest.

## Supporting information

Supplementary
