# Supplementary material for: High Discharge Energy Density at Low Electric Field Using an Aligned Titanium Dioxide/Lead Zirconate Titanate Nanowire Array
Source: Adv Sci (Weinh). 2017 Dec 27;5(2):1700512. doi: 10.1002/advs.201700512 (PMC5827564; doi:10.1002/advs.201700512)
Supplement: Supplementary file 1 — Supplementary [file ADVS-5-1700512-s001.pdf]

Copyright WILEY-VCH Verlag GmbH & Co. KGaA, 69469 Weinheim, Germany, 2017.

ADVANCED SCIENCE
Open Access

Supporting Information

for *Adv. Sci.,* DOI: 10.1002/advs.201700512

High Discharge Energy Density at Low Electric Field Using an Aligned Titanium Dioxide/Lead Zirconate Titanate Nanowire Array

*Dou Zhang, Weiwei Liu, Ru Guo, Kechao Zhou, and Hang Luo**

Copyright WILEY-VCH Verlag GmbH & Co. KGaA, 69469 Weinheim, Germany, 2016.

Supporting Information

High discharge energy density at low electric field using an aligned titanium dioxide/lead zirconate titanate nanowire array

*Dou Zhang, Weiwei Liu, Ru Guo, Kechao Zhou, Hang Luo**

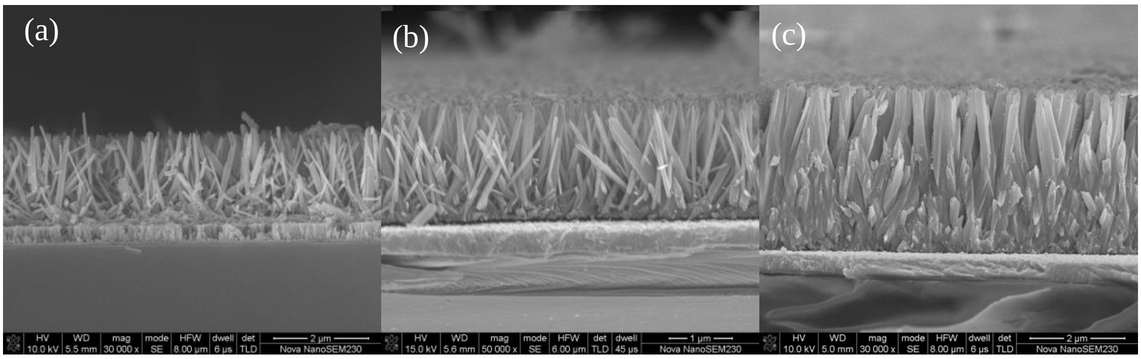

Figure S1 The morphologies of TiO_2 nanowire arrays modulated by Ti source with concentrations of (a) 0.03 mol/L, (b) 0.04 mol/L and (c) 0.06 mol/L. TiO_2 nanowire array with 0.03 mol/L. The nanowires tilt towards the substrate andbecome more and more vertical and compact with increasing Ti source concentration.

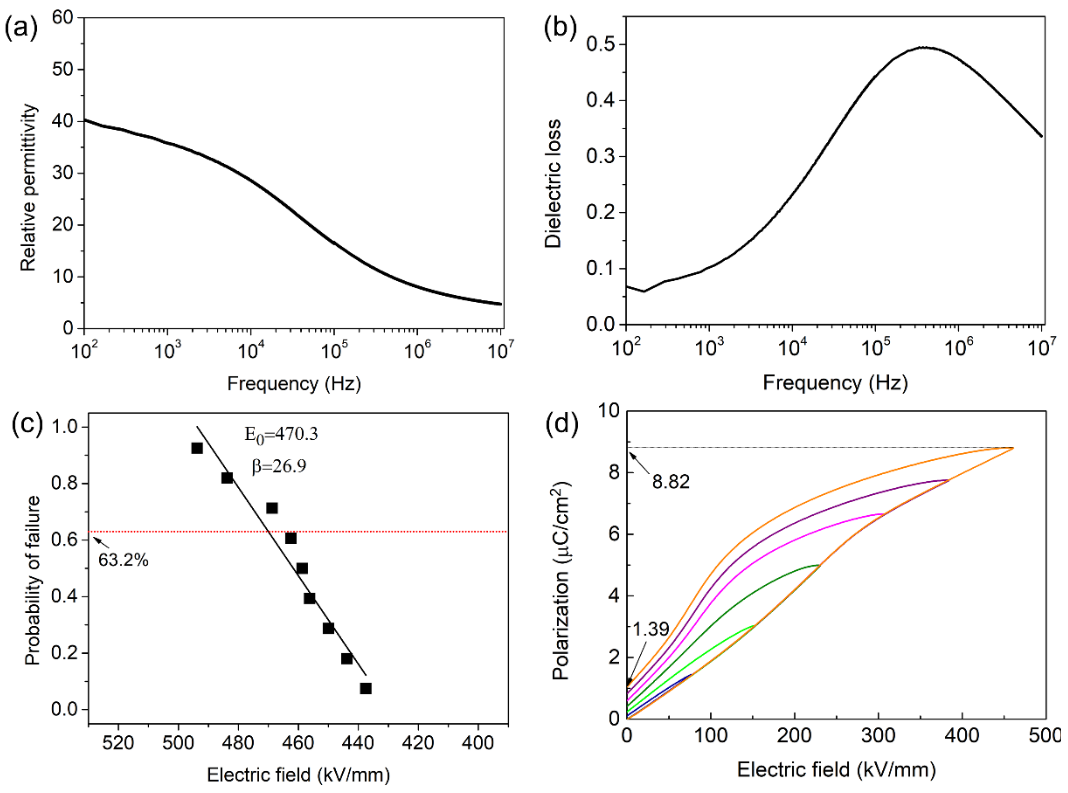

Figure S2 (a) Relative permittivity, (b) dielectric loss(c) breakdown strength, and (d) P-E loops of PVDF-TrFE-CTFE terpolymers with the mole ratio of 63:29:8. The PVDF-TrFE-CTFE terpolymers were invented by Professor Qiming Zhang at Penn State University 15 years ago and they have the highest dielectric constant among known organic polymers, therefore, they are ideal for high energy density capacitor applications. In the early studies from Professor Zhang's group, it was found that terpolymer compositions with CTFE content higher than 7 mol% have minimal remnant polarization. In these compositions, the beta phase of PVDF-TrFE can be completely converted into the alpha phase and the ferroelectric PVDF-TrFE can be converted to a ferroelectric relaxor. The ferroelectric hysteresis of PVDF-TrFE can be eliminated and the remnant polarization can be minimized.

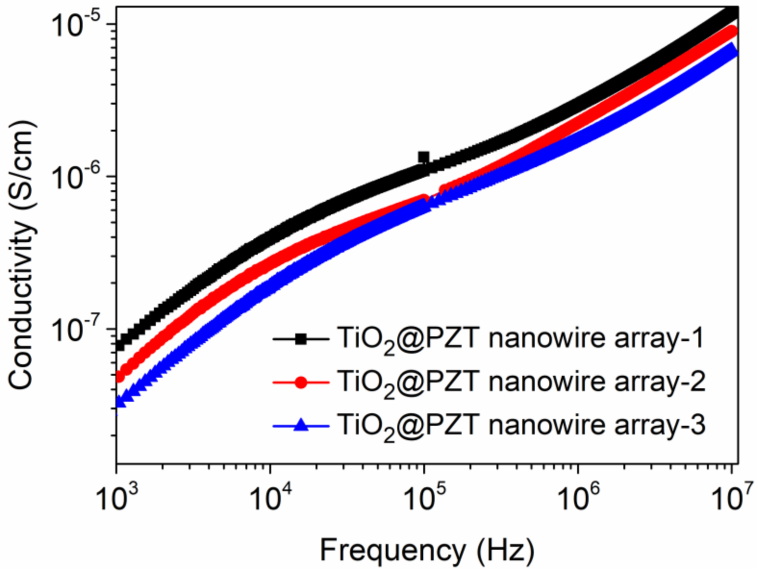

Figure S3 Frequency dependence of the electric conductivity of the nanocomposites

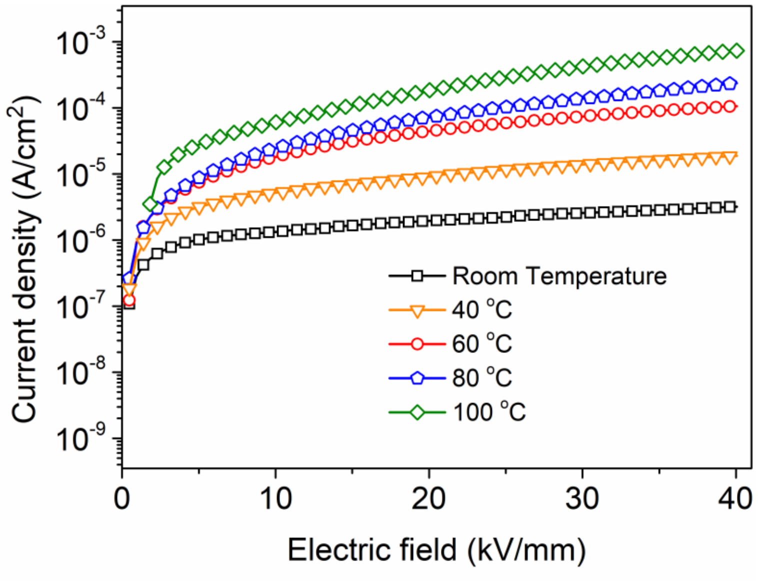

Figure S4 Leakage current density of the nanocomposites under different temperatures as a function of electric field.

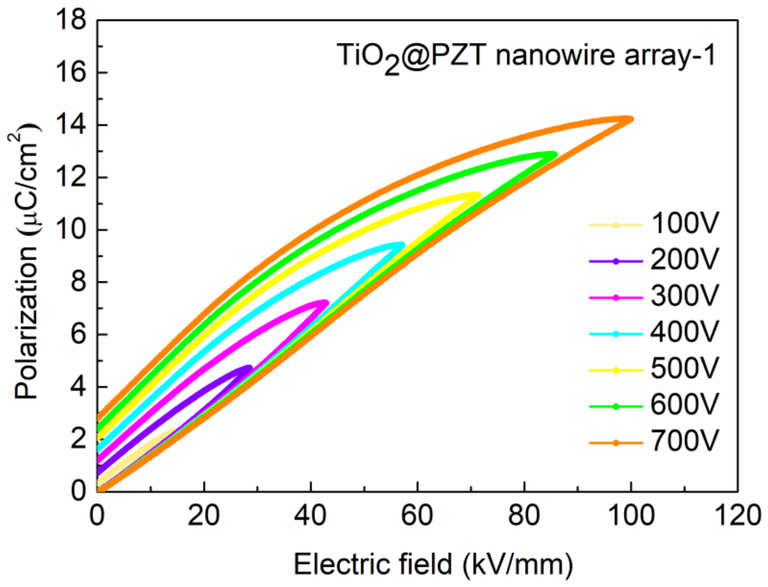

Figure S5 The *P*-*E* loops of the TiO_2@PZT nanowire array-1 nanocomposite.

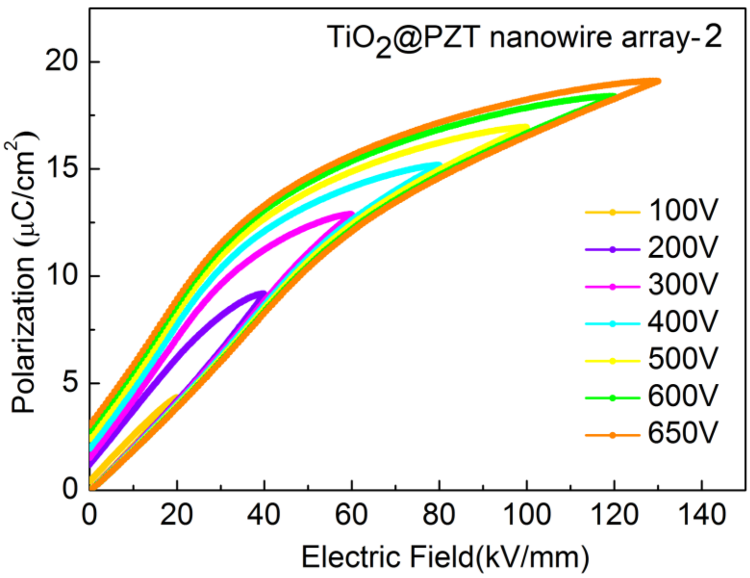

Figure S6 The *P*-*E* loops of the TiO_2@PZT nanowire array-2 nanocomposite.

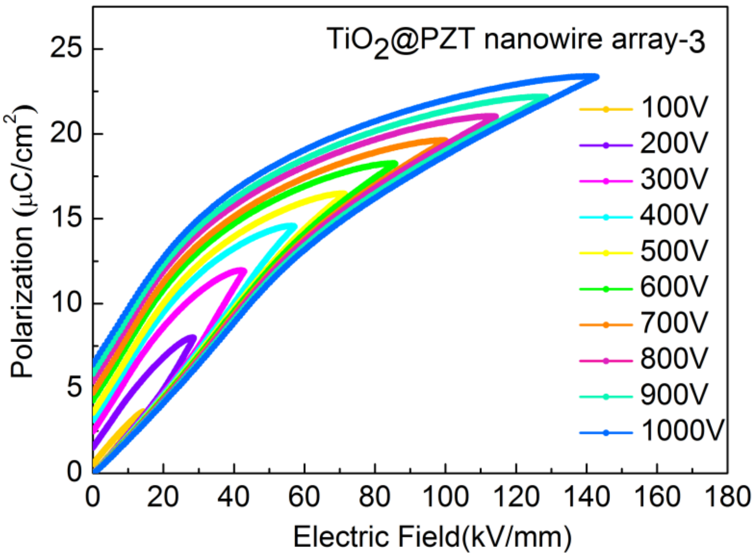

Figure S7 The *P-E* loops of the TiO_2@PZT nanowire array-3 nanocomposite.
